# Kikuchi-Fujimoto Disease: A Rare Cause of Fever in the Returning Traveller

**DOI:** 10.1155/2014/868190

**Published:** 2014-12-10

**Authors:** Matthew R. Wilson, Gordon Milne, Evangelos Vryonis

**Affiliations:** Infectious Diseases, Monklands Hospital, Monkscourt Avenue, Airdrie ML6 0JS, UK

## Abstract

*Background.* Kikuchi-Fujimoto disease (KFD) is typically a self-limited, benign illness which presents with fever and lymphadenopathy. It is rare in Caucasians, normally occurring in those of Asian descent. The aetiology is poorly understood, but it appears to be an autoimmune disorder with a possible infectious trigger. The clinical features are such that it is often mistaken for infectious diseases or malignancy. *Case Report.* Here we describe a case of a 36-year-old Asian man who presented following a recent trip to Delhi, India. He described fever, neck swelling, and arthralgia. Given his travel history an infectious cause for his presentation was presumed but multiple investigations were negative. Persistence of his symptoms led to lymph node biopsy to investigate for malignancy; surprisingly this revealed a necrotizing lymphadenitis in keeping with KFD. The patient made a full recovery with supportive treatment only. *Conclusion.* This case presented an opportunity to reflect on two common presenting complaints—fever in the returning traveller and unexplained lymphadenopathy. Both presentations have a wide range of aetiologies to consider. Although KFD is rare, it is an important diagnosis to make as it can prevent further expensive and invasive investigations, as well as potentially harmful treatments and psychological stress to the patient.

## 1. Introduction

First described in Japan in 1972 [1, 2], Kikuchi-Fujimoto disease (KFD), or histiocytic necrotising lymphadenitis, is a relatively benign and self-limited disease that classically presents with lymphadenopathy and fever. Its aetiology is poorly understood, and it is sufficiently rare that, to our knowledge, its incidence rates have not been reported, although it is known to be much more prevalent in Asian populations [3]. Indeed, a comprehensive literature review of KFD cases in 2003 described it as being “scarcely known in the western hemisphere” [4]. KFD patients are typically young, with a mean age of diagnosis of 21 years [5].

The typical presenting features of KFD (localised lymphadenopathy with constitutional symptoms such as fever and night sweats) lead to it often being mistaken for infection or malignancy. Here we describe one such case whereby a man presented having recently returned to the United Kingdom from India.

## 2. Case Presentation

A 36-year-old Asian man, who was originally from India but had lived in the UK for over 10 years, presented to our Medical Admissions Unit in February 2014. Six weeks previously he had returned from a six-week trip to Delhi, where he had been visiting family. He had received no vaccinations before travelling and had not taken malaria prophylaxis. Whilst in India he had no unwell contacts and had stayed in a large town, with no prolonged periods in rural or remote areas.

He gave a two-week history of fever, night sweats, painful knee joint, and mild shortness of breath. He had no significant past medical history. He was febrile, with a temperature of 38.2°C, and tachycardic at 105 beats/min. Blood pressure was 142/84 mm Hg, and oxygen saturation was 98% on room air. He had bilateral submandibular and cervical lymphadenopathy and bilateral parotid swelling. The lymph nodes were nontender and rubbery. Examination of his pharynx was normal, as were cardiovascular, respiratory, and abdominal examinations.

Results of investigations undertaken on admission were normal full blood and differential white-cell count but elevated inflammatory markers (C-reactive protein: 49 mg/L (reference range RR < 10 mg/L); erythrocyte sedimentation rate: 70 mm/h (RR < 18 mm/h)). Renal and liver function tests were normal. Serum lactate dehydrogenase (LDH) was elevated at 721 U/L (RR < 280 U/L). Three separate blood samples were negative for a rapid malarial parasite test, and no malaria parasites were seen on blood film examination. Chest radiograph, urinalysis, and electrocardiograph were unremarkable.

The initial impression was of either mumps or a travel-related tropical disease. He was treated as having sepsis of unknown origin with broad-spectrum intravenous antibiotics whilst further investigations were carried out. Two salivary samples taken one week apart were negative for mumps immunoglobulin M. Multiple blood and urine cultures were sterile. Viral serology for HIV and hepatitis A, B, and C and for cytomegalovirus was negative. Syphilis and dengue fever serology were negative. Three early-morning urine samples for acid-alcohol fast bacilli were negative. A “Monospot” test for infectious mononucleosis was negative. Serum immunoglobulin values were normal, and anti-nuclear antibody screening was negative.

**Figure 1:**
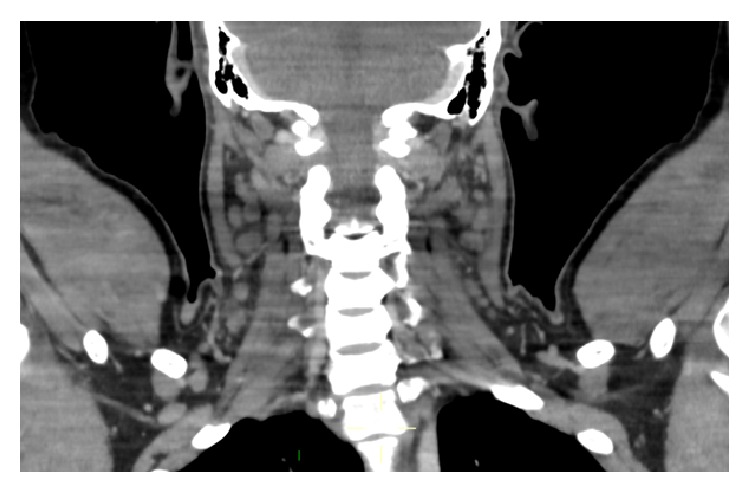
Computer tomographic image demonstrating multiple bilateral enlarged cervical lymph nodes.

Over the next two weeks, episodic fever continued. Antibiotics were stopped after one week as they had conferred no symptomatic benefit or improvement in blood inflammatory markers. Paracetamol and nonsteroidal anti-inflammatory agents were given for symptomatic relief. His cervical lymphadenopathy persisted. Ultrasound scanning revealed several prominent hypoechoic lymph nodes throughout the neck and prominent lymph nodes in the groin. Supported by the elevated LDH value, the clinical impression at this point was that of lymphoma. Computer tomographic imaging of the neck, chest abdomen, and pelvis revealed bilateral enlarged cervical lymph nodes (Figure 1) but no other significant abnormality.

**Figure 2:**
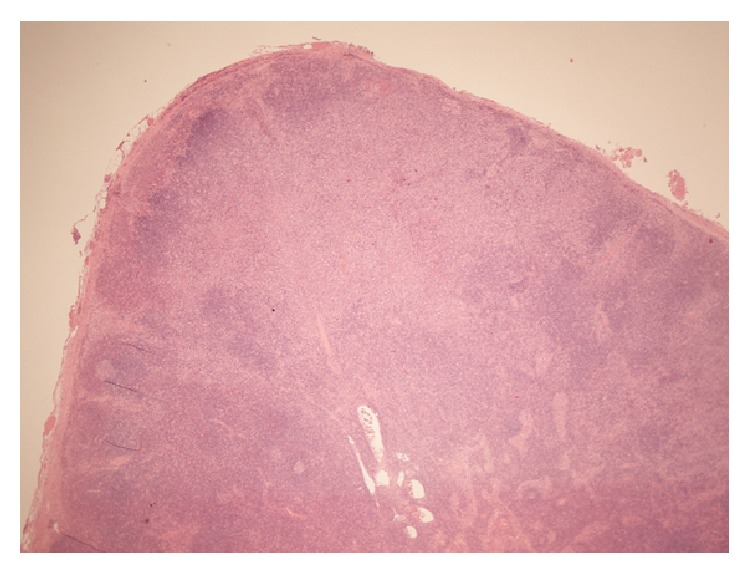
Low power (×40) view showing a large area of paracortical necrosis (Hematoxylin and Eosin stain).

**Figure 3:**
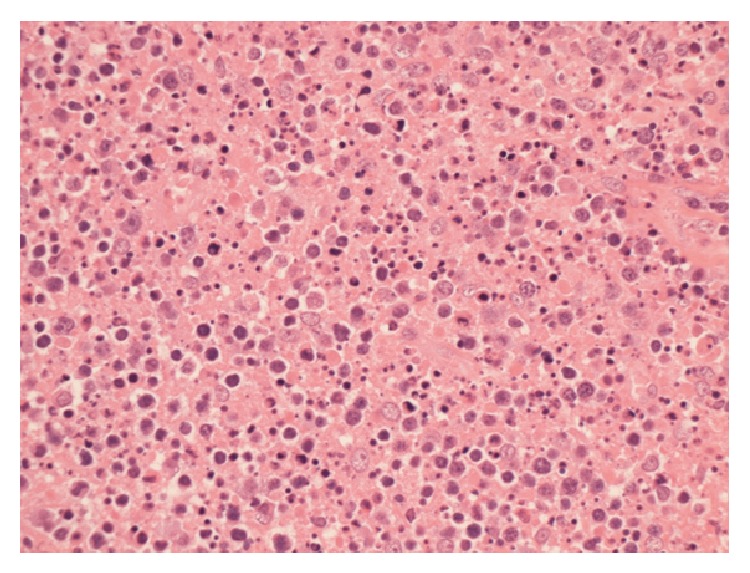
High power (×400) view showing individual cell death and nuclear debris (karyorrhexis) (Hematoxylin and Eosin stain).

An excisional biopsy of a cervical lymph node revealed reactive hyperplasia and areas of necrosis with abundant apoptotic debris. Polymorphonuclear leukocytes and plasma cells were not readily identified, but numerous monocytoid cells were present (Figures 2 and 3). The overall appearance was of a necrotising lymphadenitis, in keeping with KFD.

When the diagnosis was made, four weeks after presentation, the patient was feeling better, with resolution of fever and night sweats and almost complete reduction in the cervical lymphadenopathy. Two months later he remained well, with no relapse in symptoms.

## 3. Discussion

KFD was discovered in 1972 in Japan, when it was reported independently by two separate groups [1, 2]. Its exact incidence is unknown, but it is more prevalent in East Asia and the Far East [6]. Traditionally, it was felt to have a strong female preponderance (male : female ratio of 1 : 4), but recent reports have challenged this opinion and suggest that the actual ratio is closer to 1 : 1 [7]. KFD is often thought of as a disease of the young, with a mean age of diagnosis of 25 reported in an analysis of 244 cases [6]. However, it has also been reported in patients ranging from 6 to 80 years old, most of whom were previously well [8–10].

The aetiology of KFD remains unknown. The geographic predominance in Asian countries may be related to the presence of the HLA class II alleles,* HLA-DPA1* and* HLA-DPB1*, which are prevalent in Asian KFD patients but extremely rare among Caucasians [11], an observation perhaps reflective of a genetic predisposition to the disease. An infectious cause, or at least trigger, to KFD has been postulated. Organisms such as Epstein-Barr virus, cytomegalovirus, varicella-zoster virus, human herpes virus-6, human immunodeficiency virus,* Yersinia enterocolitica*, and* Toxoplasma gondii* have been implicated, but no convincing causal relationship has been identified [6]. Some authors have hypothesised that KFD is a self-limited autoimmune condition triggered by virus-infected transformed lymphocytes [12]. This theory is based on the histopathological features of the disease, which are similar to those seen in viral infections [4]. However, serological testing and histological staining for viruses have been consistently unhelpful in supporting this theory.

In many reported cases, KFD has coincided with, preceded, or followed a diagnosis of systemic lupus erythematosus (SLE); 32 of 244 KFD patients in one series also had SLE. Although SLE may be more prevalent in KFD patients, a clearly defined relationship between the two conditions has not been identified. Care should be taken in making the diagnosis of concomitant SLE in the patient with KFD as the two diseases share some clinical features (lymphadenopathy, rash, pyrexia of unknown origin, and arthralgia). Other autoimmune diseases such as Still's disease, Sjögren's syndrome, polymyositis, and rheumatoid arthritis also have been reported as occurring in conjunction with KFD, albeit with less frequency than SLE [12].

KFD typically is a self-limited condition, with duration of 1 to 4 months, although a recurrence rate of 3-4% has been reported [13]. The most common presenting signs are cervical lymphadenopathy (70–80%) and fever (30–50%) [3]. Other less frequently reported symptoms are fatigue, arthralgia, rash, and weight loss. Affected lymph nodes are typically painless, solid, and mobile [14]. Although cervical nodes are most often affected, nodes in other regions, including the axilla and groin, can be involved. Hepatomegaly and splenomegaly have been reported in isolated cases, 3% and 2%, respectively, in an analysis of 244 cases in 2007 [6].

Routine laboratory indices are typically unhelpful in establishing a diagnosis of KFD. Reported haematological findings are leukopenia, neutropenia, lymphocytosis, thrombocytopenia, or anaemia [3]. Notably, our patient displayed none of the above abnormalities. Biochemical abnormalities reported in other cases are elevated inflammatory markers (C-reactive protein and erythrocyte sedimentation rate), elevated liver transaminases, increased LDH, antinuclear antibody positivity, and reduced complement 3 values [6]. Our patient had raised inflammatory markers and LDH only, findings which may be present in many infectious or malignant processes.

Imaging, such as computed tomography or magnetic resonance imaging, is unhelpful in distinguishing the lymphadenopathy of KFD from other causes. These imaging tests are often performed, however, to identify a suitable site for lymph node biopsy or excision.

The list of differential diagnoses for our patient, and for KFD in general, is long and includes any potential cause of lymphadenopathy and fever. Infectious causes are likely to be considered first, particularly as in our patient who had recently travelled to India. We considered tuberculosis, toxoplasmosis, human immunodeficiency virus, Epstein-Barr virus, herpes simplex, dengue fever, and mumps. As discussed above, autoimmune conditions such as SLE can cause a presentation similar to KFD and are therefore high in the differential diagnosis. Finally, malignancy (both haematological and solid-organ) is undoubtedly the most important diagnosis to exclude in a patient presenting with features of KFD. The high LDH levels and lymphadenopathy in our patient made us highly suspicious of lymphoma, and the excisional lymph node biopsy was carried out to exclude or confirm that diagnosis.

The definitive diagnosis of KFD can be made only through lymph node biopsy and histological examination [5]. Even with adequate tissue the lymph node appearances can be mistaken for malignant lymphoma; in one study, 30% of lymph node biopsies in KFD were initially misdiagnosed as lymphoma [15]. The histopathological features of KFD have been classified into three stages: (1) proliferative stage, with expression of histiocytes, plasmacytoid monocytes, and lymphoid cells containing karyorrhectic nuclear fragments and eosinophilic apoptotic debris; (2) necrotising stage, with a degree of coagulative necrosis; and (3) xanthomatous stage, with foamy histiocytes predominating [5, 16]. A histological analysis in 2004 disputed this theory slightly, suggesting that the xanthomatous stage is not the resolving stage of KFD but is a histological variant of KFD in its own right [17]. A characteristic and useful diagnostic feature is the absence of granulocytes in the “necrotising stage”, which is helpful in distinguishing KFD from SLE and drug induced lymphadenopathy.

Since KFD is a self-limited illness, often no treatment is required. Supportive measures, including nonsteroidal anti-inflammatory drugs and antipyretics, can be used for relief of lymph node tenderness, arthralgia, and fever. Corticosteroids are generally reserved for severe cases, or where supportive measures fail to control symptoms [4]. Other immunosuppressive agents (hydroxychloroquine, cyclosporine, and azathioprine) have been used successfully in individual cases. The course of the illness is usually about 1–3 months, but longer follow-up may be appropriate as a 4% relapse rate has been reported [5], and monitoring for the development of SLE is prudent.

One analysis of 244 cases of KFD reported an overall mortality rate of 2.1%, which contradicts the widely held belief that the disease is generally benign and nonfatal. A female patient died of heart failure, in the context of other haematological autoimmune complications including haemolytic anaemia. Another patient died of pulmonary haemorrhage and a 24-week pregnant woman died from multiorgan failure after developing KFD-triggered hemophagocytic syndrome. Three patients developed KFD after organ transplantation and died of respiratory failure. These patients were immunosuppressed and there were probably other factors than KFD involved in their illness [6].

## 4. Conclusion

This case describes an unusual and unexpected cause of fever and lymphadenopathy in a returning traveller. We believe that the initial focus on infectious causes was the correct approach in this patient, but the case illustrates that a history of foreign travel can sometimes be coincidental rather than directly implicated in such a presentation. It should also be remembered that the approach to the febrile traveller should not simply focus on potential exposure to infectious diseases, but also on the ethnicity of the patient, which can make the patient more or less predisposed to certain conditions. Kikuchi-Fujimoto disease is rare and relatively benign, but its clinical features can easily be mistaken for more sinister diseases. Establishing the diagnosis can therefore prevent further expensive and invasive investigations, as well as potentially harmful treatments and psychological stress to the patient.
